# Hyaluronic Acid Rectal Wall Infiltration and Reversal in Patients With Prostate Cancer Undergoing Radiation Therapy–A Case Series

**DOI:** 10.1016/j.adro.2026.102119

**Published:** 2026-06-20

**Authors:** Zakaria El Kouzi, Ramez Kouzy, Asif Aziz, Michael K. Rooney, Stephen R. Lee, Stephen E. McRae, Ketan Y. Shah, Mohamed E. Abdelsalam, Ethan Y. Lin, Comron Hassanzadeh, Seungtaek Choi, Quynh-Nhu Nguyen, Steven J. Frank, Mohamed Shelan, Lauren L. Mayo, Sean McGuire, Steven Yevich, Osama Mohamad

**Affiliations:** a Department of Genitourinary Radiation Oncology, University of Texas MD Anderson Cancer Center, Houston, Texas; b Department of Radiation Oncology, University of Texas MD Anderson Cancer Center, Houston, Texas; c Department of Interventional Radiology, University of Texas MD Anderson Cancer Center, Houston, Texas; d Department of Radiation Oncology, Inselspital Bern, University of Bern, Bern, Switzerland

## Introduction

Radiation therapy (RT) is a standard definitive treatment offered for many of the 300,000 new cases of prostate cancer diagnosed in the United States every year.^1^ Advancement in RT technologies, such as intensity modulated RT, image guidance, and improved dose conformality have allowed greater adoption of hypofractionated treatment courses in as little as 5 fractions. However, greater dose per fraction can increase the risk for rectal toxicity.

One method to decrease risk of radiation damage to the rectal wall is through injection of an inert gel into the fat plane between the prostate and rectum. The gel acts as a spacer, effectively providing a space between the 2 organs. The injection is performed through a needle in the outpatient setting using a transperineal approach with transrectal ultrasound for image guidance. Patients are often under moderate or deep sedation. Placement of fiducial markers in the prostate can be performed in the same procedural setting for subsequent radiation planning. After injection of the gel, the patient proceeds with pelvic computed tomography and/or magnetic resonance imaging (MRI) for radiation planning purposes and then radiation according to this plan. The gel naturally resorbs completely in 3 to 12 months.

Randomized trials have demonstrated the protective effects of these rectal spacers against low-grade rectal adverse events.^2^, ^3^, ^4^ These data led to an explosion in the use of rectal spacers in the United States. However, placement of spacers can lead to potentially serious, although rare, adverse events such as rectal ulceration or perforation when the gel advances from the fat plane to dissect into the anterior rectal wall.^5^^,^^6^ Careful consideration of the risks of spacer placement is critical for optimal shared decision making. Development of risk mitigation strategies of spacer placement and management of complications could improve the therapeutic ratio for patients with prostate cancer receiving RT.

At our institution, rectal spacers are often injected in conjunction with placement of prostate fiducial marker. All procedures are performed by an interventional radiology faculty with at least 5 years of experience. Two different gels are used: SpaceOAR (Boston Scientific) and Barrigel (Teleflex). Selection of which gel to inject is determined at the time of procedure scheduling at the discretion of the radiation oncologist. After the procedure, planning MRI or computed tomography is performed before radiation.

In the event of rectal wall infiltration, discussion ensues between the radiation oncologist and the interventional radiologist on the next best course of action. If SpaceOAR gel has infiltrated the rectal wall, no radiation is performed until the gel has completely resorbed based on imaging, which typically requires 3 to 6 months of follow-up. If Barrigel has infiltrated the rectal wall, then there is the potential need for injection of a reversal agent, hyaluronidase. There is a paucity of literature on the gravity of Barrigel infiltration and the indication for injection of the reversal agent. At the time of submission, only 1 case report had been published in the literature.^7^

In this report, we present 5 cases of suspected rectal wall infiltration from hyaluronic acid (HA) injection (Barrigel) of 803 patients who received the gel for prostate cancer between September 2023 and December 2025. For each patient, we outline the clinical history, images of spacer placement, treatment strategies including use of hyaluronidase enzyme whenever deemed appropriate, and overall clinical outcome. We conclude by providing recommendations for diagnostic and management strategies.

## Patient Cases

### Case 1

**Figure 1:**
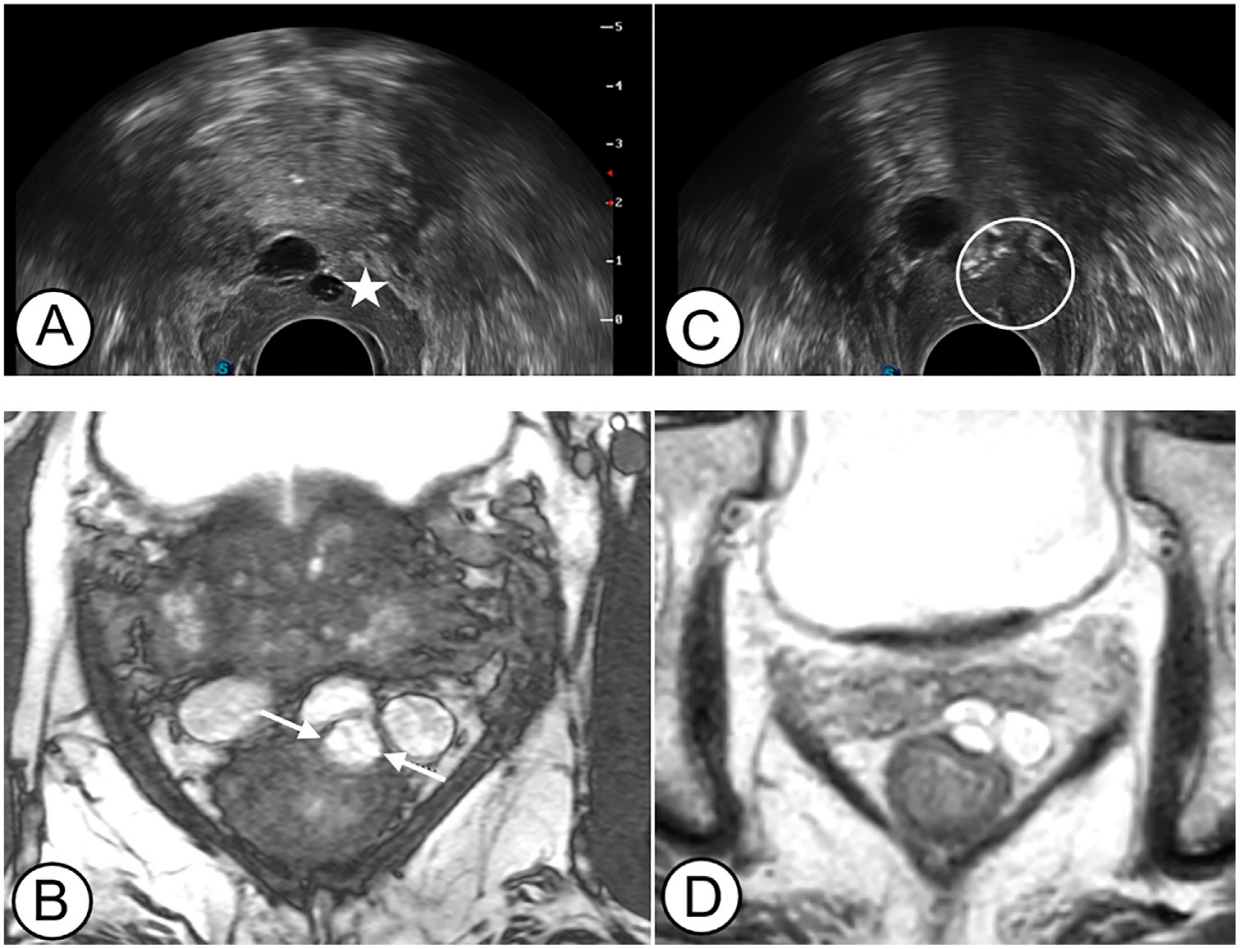
(A) Ultrasound imaging at the time of the procedure showing a small amount of gel infiltrating the rectal wall (star). (B) MRI images confirm rectal wall infiltration. (C) Ultrasound imaging immediately after hyaluronidase enzyme injection showing the complete resolution of small infiltrated hyaluronic acid (circle).(D) MRI simulation after hyaluronidase showed the resolution of the small infiltration, with persistent hyaluronic acid in the space between the prostate and rectum. *Abbreviation:* MRI = magnetic resonance imaging.

A 77-year-old man with National Comprehensive Cancer Network (NCCN) favorable intermediate-risk prostate cancer (Gleason score 7 = 3 + 4, prostate-specific antigen [PSA] 3.5) opted for stereotactic body RT (SBRT). An HA gel was administered under transrectal ultrasound guidance, but the injection was stopped at 7.5 mL when a small amount of spacer was suspected to dissect into the rectal wall based upon procedural rectal ultrasound imaging (Fig. 1A). The patient had no rectal pain or other pertinent symptoms. To confirm or rule out rectal wall infiltration, flexible sigmoidoscopy was performed 8 days later but did not show evidence of rectal thickening or rectal perforation. A diagnostic MRI confirmed rectal wall infiltration (Fig. 1B). Due to the uncertainty of whether to proceed with RT in the presence of infiltration, a multidisciplinary decision was made to proceed with injection of reversal agent. Approximately 1 month after the initial procedure, hyaluronidase (200 **U**) was injected to dissolve the small aliquot of spacer that had infiltrated into the rectal wall submucosa, while leaving the gel appropriately positioned between the rectum and prostate untouched (Fig. 1C). A treatment planning MRI confirmed resolution of the infiltration (Fig. 1D). Throughout this period, the patient remained asymptomatic. The patient received SBRT as planned (36.25 Gy in 5 fractions)**.** Fourteen months later, the patient had no gastrointestinal (GI) symptoms.

### Case 2

**Figure 2:**
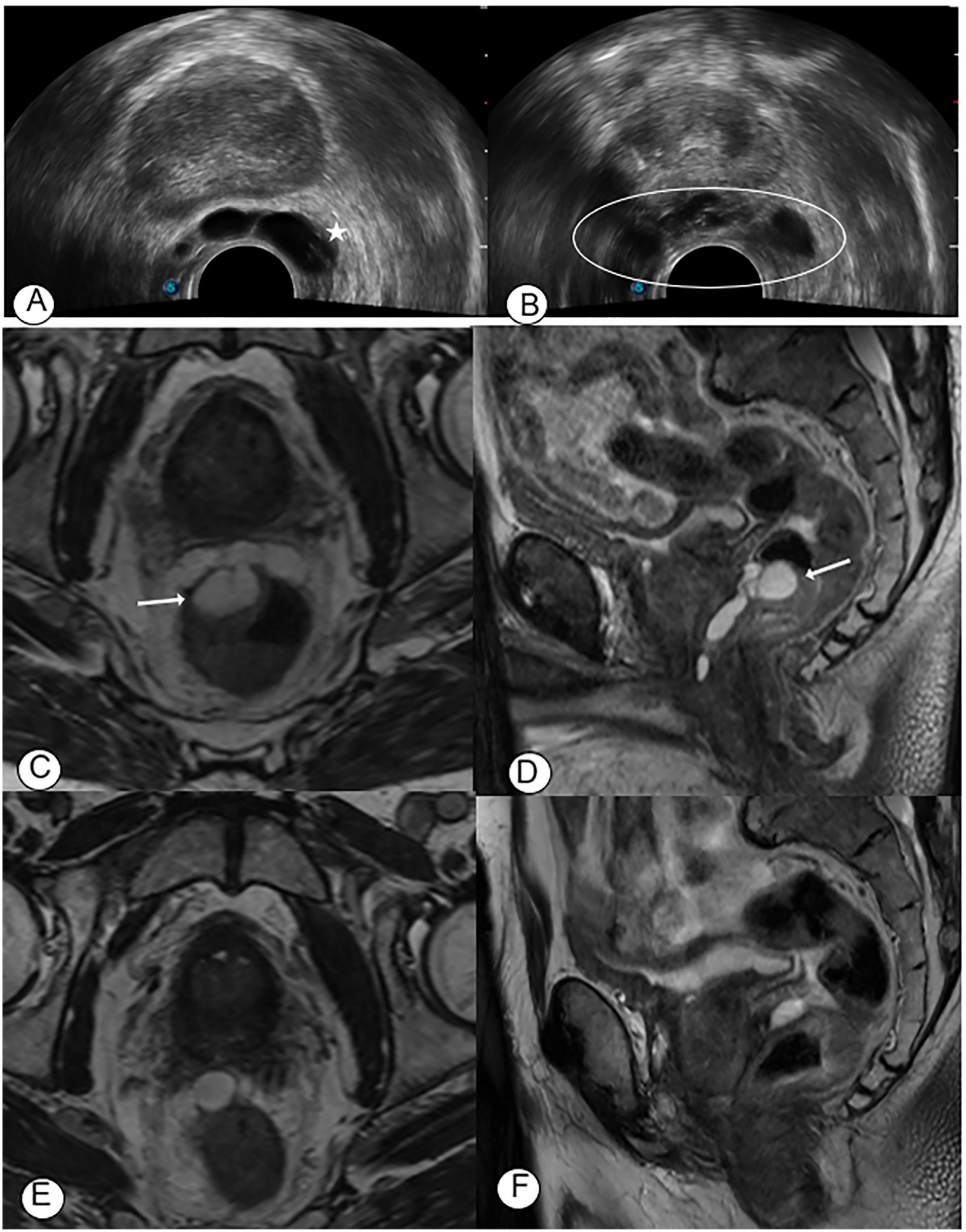
(A) Ultrasound imaging at the time of the procedure showing spacer gel infiltrating into the rectal wall (star). (B) Ultrasound imaging after hyaluronidase was used showing the resolution of rectal infiltration (circle). (C, D) Diagnostic MRI scan showing rectal wall infiltration (white arrow). (E, F) Treatment planning MRI scan showing resolution of rectal wall infiltration after hyaluronidase injection. *Abbreviation:* MRI = magnetic resonance imaging.

A 71-year-old man with NCCN high-risk prostate cancer (Gleason 9, PSA 6.3) was recommended RT and underwent rectal spacer injection (9 mL). Intraprocedural imaging showed some spacer gel tracking laterally, concerning for possible rectal wall infiltration (Fig. 2A). The patient was asymptomatic. A diagnostic MRI showed rectal wall infiltration (Figs. 2C, D), and 450 units of hyaluronidase were injected to dissolve the spacer specifically within the rectal wall, while leaving the gel between the rectum and prostate untouched (Fig. 2B). A follow-up MRI confirmed resolution of the spacer material that had previously been seen in the rectal wall (Fig. 2E, F). The patient completed RT (70 Gy in 28 fractions) and had no major GI adverse events at last follow-up (4 months).

### Case 3

**Figure 3:**
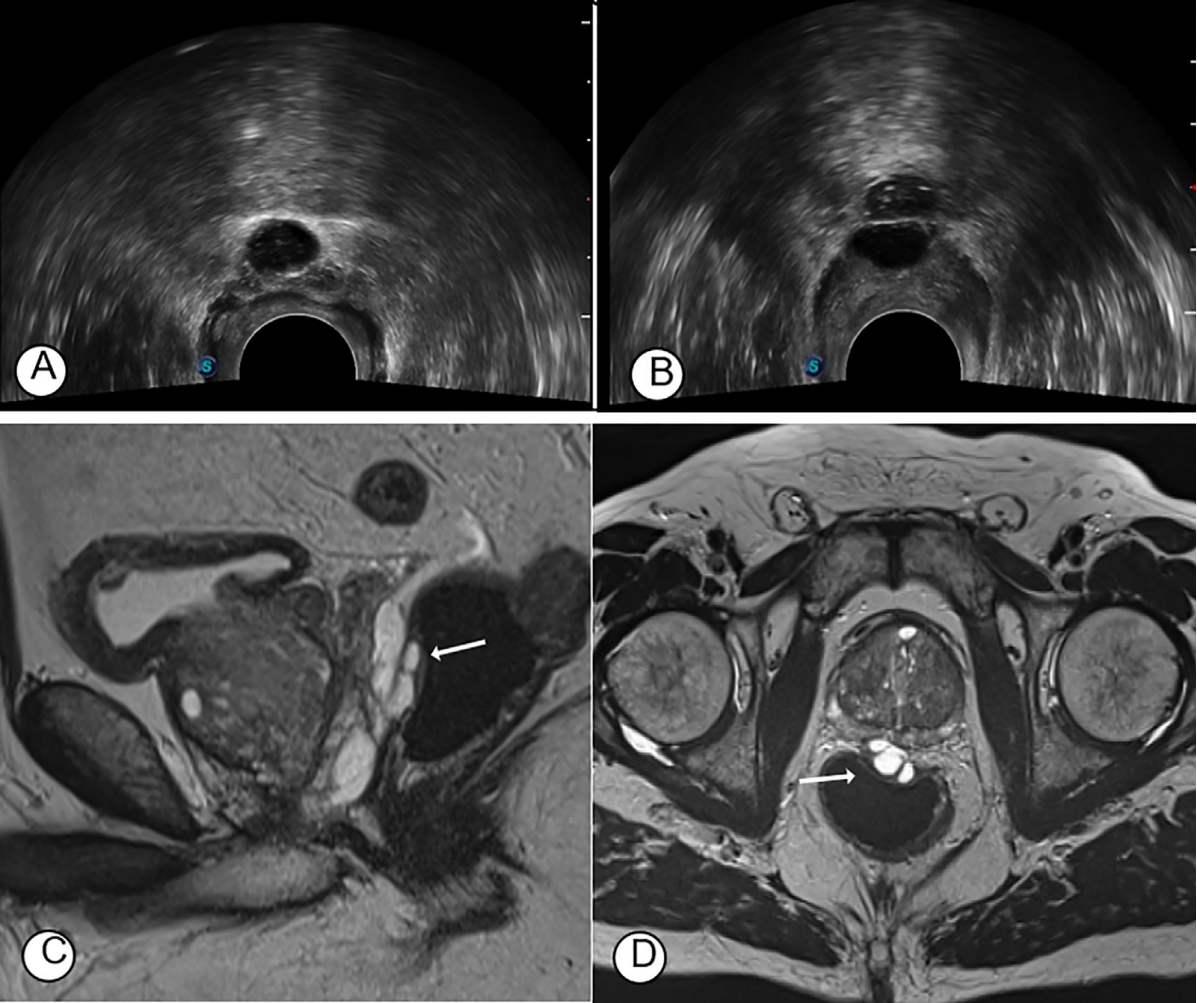
(A, B) Ultrasound imaging at the time of the procedure showing no obvious signs of the gel infiltrating the rectal wall. (C, D) Treatment planning magnetic resonance imaging scan showing potential signs of minor rectal wall infiltration (white arrow).

A 73-year-old man with NCCN favorable intermediate-risk prostate cancer (Gleason 6, PSA 11) elected for definitive proton therapy. A rectal spacer was placed (9 mL). During the procedure, the physician was not concerned for infiltration based on transrectal ultrasound images (Fig. 3A, B). Treatment planning MRI demonstrated possible small rectal wall infiltration (Fig. 3C, D). The patient remained completely asymptomatic. Given absence of symptoms and the small questionable rectal wall infiltration, a multidisciplinary panel decided to proceed with proton therapy (76 Gy in 38 fractions). The patient completed RT and had no major side GI adverse events at last follow-up (6 months).

### Case 4

**Figure 4:**
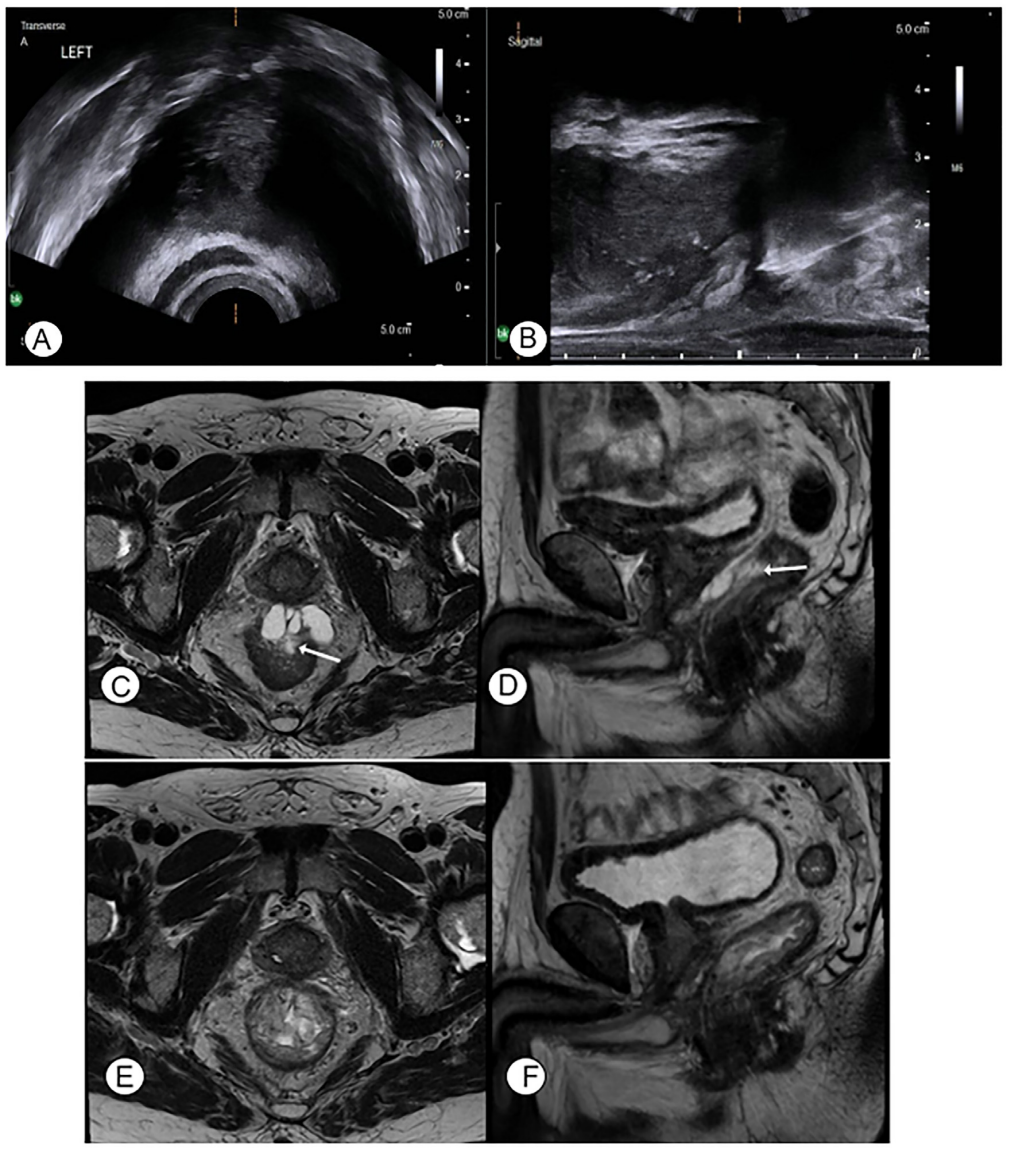
(A, B) Ultrasound imaging at the time of the procedure. (C, D). Treatment planning MRI scan showing rectal wall infiltration (white arrow). (E, F) Repeat treatment planning MRI scan showing resolution of rectal wall infiltration after hyaluronidase injection. *Abbreviation:* MRI = magnetic resonance imaging.

A 69-year-old man with NCCN unfavorable intermediate-risk prostate cancer (PSA 18.1, Gleason 4 + 3) was recommended RT. The patient underwent rectal spacer insertion (9 mL) with no concerning findings on procedural transrectal ultrasound (Fig. 4A, B). Postprocedural treatment planning MRI showed rectal wall infiltration (Fig. 4C, D). The patient remained asymptomatic. One week later, 1650 units of hyaluronidase were injected to dissolve the spacer, and MRI (Fig. 4E, F) confirmed dissolution of the rectal spacer. The patient completed proton therapy (70 Gy in 28 fractions) and had no major GI adverse events at last follow-up (2 months).

### Case 5

**Figure 5:**
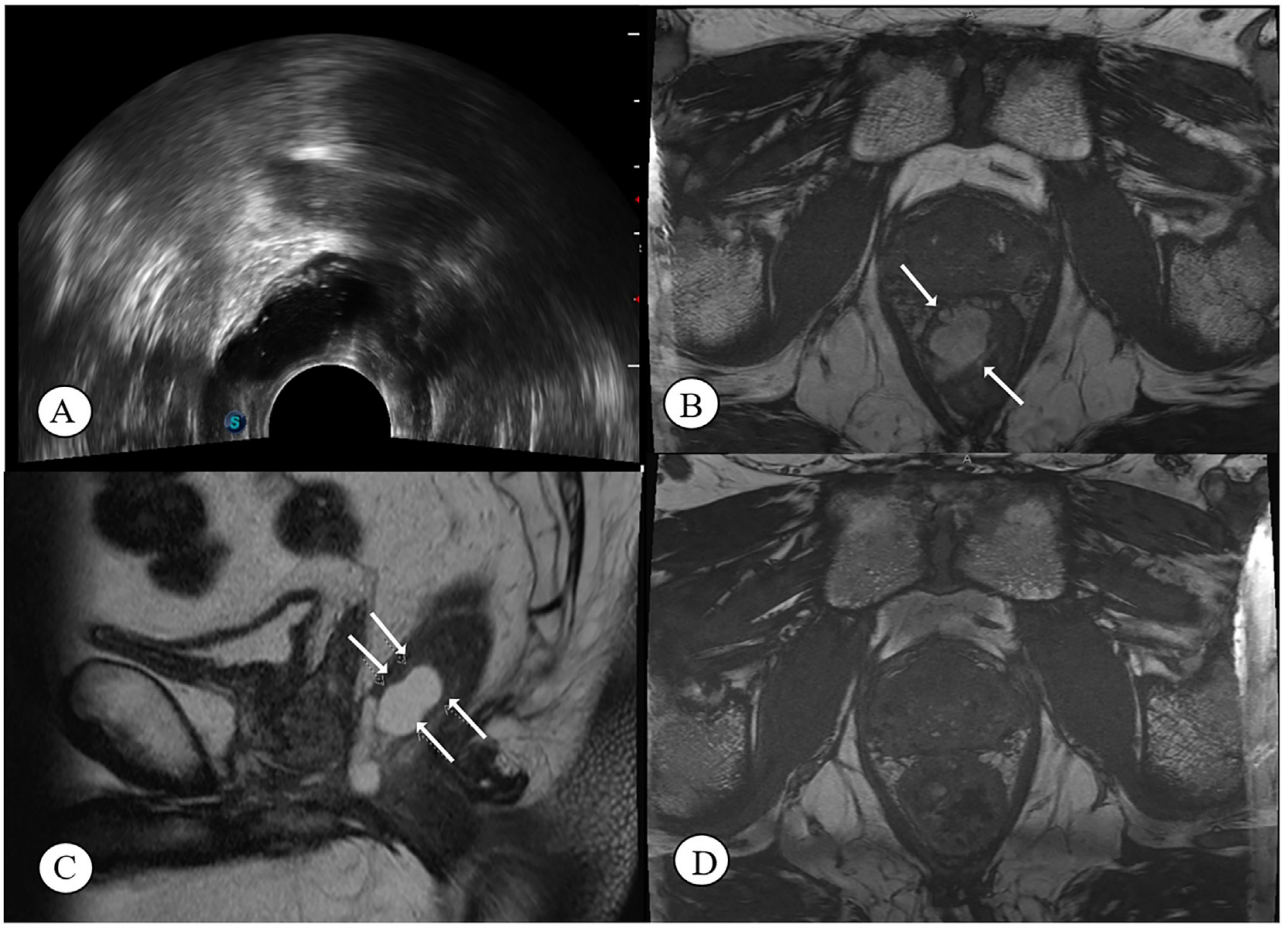
(A) Ultrasound imaging at the time of the procedure. (B, C) Diagnostic MRI showing rectal wall infiltration (white arrows). (D) Treatment planning MRI showing resolution of rectal wall infiltration after hyaluronidase injection.

A 65-year-old man with favorable intermediate-risk prostate cancer (Gleason 6, PSA 18) treated with RT (intensity modulated RT to 75.6 Gy) in 2014 had biochemical failure in 2025. Transrectal prostate biopsy showed a local recurrence on the right side. The patient opted for partial gland salvage high-dose-rate brachytherapy. Post-RT fibrosis complicated the HA gel insertion (Fig. 5A). Postprocedural MRI planning showed extravasation of gel into the rectal wall (Fig. 5B, C), and 600 units of hyaluronidase were injected to dissolve it. Follow-up MRI (Fig. 5D) show resolution of the spacer. The patient completed his high-dose-rate brachytherapy using Ir-192 (13.50 Gy × 2 fractions) and remains without GI symptoms 1 month after the procedure. All the cases are summarized in Table 1.

**Table 1:** Fischer-Valuck RWI grading, volume of misplaced Barrigel, volume of hyaluronidase used, and symptoms of all cases

Cases	RWI grading	Volume misplaced Barrigel (mL)	Dimensions misplaced Barrigel (cm) (ml × ap × cc)	Volume of hyaluronidase injected (mL)	Dose of hyaluronidase (U)	Symptoms
Case 1	2	0.4	0.6 × 0.5 × 1.3	1	200	Asymptomatic
Case 2	2	2.8	1.0 × 2.0 × 1.4	10	450	Asymptomatic
Case 3	2	0.8	0.7 × 0.7 × 1.7	None	None	Asymptomatic
Case 4	2	8	1.9 × 1.7 × 2.5	11	1650	Asymptomatic
Case 5	2	9.7	1.4 × 2.3 × 3.1	4	600	Asymptomatic

*Abbreviations:* ap = anterior-posterior; cc = cranio-caudal; ml = medio-lateral; RWI = rectal wall infiltration.

## Discussion

Commonly used rectal spacers carry a small risk of significant toxicity including rectal perforation.^5^^,^^6^ These rare complications may impair the patient’s quality of life and require aggressive salvage treatments including surgical resection with colostomy. Thus, early recognition of infiltration is critical.^8^ With the rise in rectal spacer use, the likelihood of complications may increase. Here, we report 5 cases for which there was concern of rectal infiltration after HA spacer placement on ultrasound or MRI, with a detailed description of our diagnostic and management approach. In 4 of our cases, hyaluronidase injection was performed, whereas in the remainig case, we monitored with imaging before proceeding with RT. From this experience, we highlight several recurrent challenges and identify opportunities for improvement.

### Our institutional approach to HA gel insertion

At our institution, we have developed a standardized collaboration between radiation oncology and interventional radiology in which a board-certified interventional radiologist performs the injections with the patient under general anesthesia in a routine sterile setting according to the manufacturers’ guidelines. In brief, an 18-G needle is advanced via the perineum into the fat plane between the prostate and rectum under transrectal ultrasound guidance. The gel is then slowly administered up to 9 mL, but the total volume is modified at the discretion of the proceduralist based on prostate size and ease or difficulty of injection. Attempts are made to evenly distribute the spacer gel especially at the prostate apex. In the event of fibrosis or scarring, due to prior RT for example, attempts may be made by the operator to reposition the needle in different aspects of the fat plane to improve dissection. The gel might infiltrate into the anterior rectal wall if the access needle penetrates the rectal wall upon insertion. Prior trauma to the rectal wall from transrectal prostate biopsies, prior RT, or infections may increase risk for infiltration. When rectal wall infiltration is identified, the challenge falls upon the interdisciplinary team to determine the best course of action for the patient. Naturally, there is concern to proceed with RT in the presence of rectal wall infiltration as this might increase the risk for rectal wall damage, particularly considering the post-RT inflammatory response.

### Diagnostic challenges

Several proceduralists that are experienced with HA injection have noted that Barrigel rectal spacers often appear as large ‘lumps’ of gel. This formation appears to be more prominent if acute inflammation or chronic fibrotic scarring is present between the prostate and rectum at the time of injection. Although inherently benign, the lumped formation may provide the false impression of infiltration on MRI when the rectum, a hollow supple organ, collapses and envelopes around the lump of gel. Thus, an MRI might suggest infiltration even when the rectal wall is intact. As such, we advise that clinicians correlate suspicious MRI findings with intraprocedural ultrasound images and patient symptoms. The intact appearance of the muscularis propria on MRI sequences should reduce overcalling infiltration. Multidisciplinary collaboration is therefore very helpful for diagnostic accuracy and might require additional imaging with transrectal ultrasound or contrast-enhanced MRI. Prostate MRI T2 sequence appears optimal to identify small rectal infiltrations. In questionable cases of infiltration, a transrectal ultrasound may be helpful to discern actual presence and degree of very small infiltration. It remains uncertain whether very small rectal wall infiltration (<0.5 mL) exposes the patient to risk should radiation be pursued without reversal by injection of hyaluronidase.

### Management strategies

Although rectal infiltration can theoretically increase the risk of serious rectal toxicity, actual complications appear to be extremely rare—particularly when the infiltration is small, or ‘partial,’ and not full-thickness. Case 1 was our first group experience with rectal infiltration with HA. The injection of the reversal agent, hyaluronidase, was performed out of abundant precaution. In retrospect, with our subsequent experience, the reversal might not have been required for such a small rectal wall infiltration. Indeed, in a later case (case 3), we elected to proceed with RT despite the presence of small infiltration, without any adverse events post-RT. Now, our interdisciplinary team leans toward observation for minimal or equivocal infiltration, as long as the patient remains asymptomatic and imaging does not show a full-thickness involvement or active inflammation or infection. However, we do maintain the need for vigilance as even a small infiltration may be a cause of concern for the radiation oncologist when planning high-dose treatments such as SBRT or brachytherapy. In these cases, we have found that additional imaging may be needed to ensure stability of the suspected infiltration.

Larger infiltrations of HA within the rectal wall will naturally pose more concern to the multidisciplinary group. In the other 3 cases of hyaluronidase injection, the reversal agent was administered as the infiltration was deemed to be substantial or definite. Although the patients remained asymptomatic, and there were no indications for inflammation or infection on the MRI scans, the radiation oncologist naturally was more concerned for RT complications. The ability to reverse and remodel HA gel offers significant benefits, giving the treating physician and the patient more comfort and limiting significant treatment delays. Other types of spacer material may not be reversible, and the patient is usually recommended to delay treatment start by several months while the spacer dissolves naturally.^9^ When compared to polyethylene glycol spacers, HA gels are softer (which reduces risk of ischemia), are reversible with hyaluronidase, and they allow real-time adjustments during placement. We thus recommend that the decision of which rectal spacer to select should take into consideration the formulation of the gel and reversal options.

The use of hyaluronidase for rectal spacers has been reported previously, but to our knowledge, this is the first case series report in the United States. Hong et al^7^^,^^10^ from Australia documented a case of HA infiltration and reversal using hyaluronidase. Vanneste et al^11^ studied hyaluronidase in vitro and found that higher concentrations of the enzyme significantly dissolved HA, which is particularly useful when HA is mistakenly inserted into the rectal wall. Lower doses of hyaluronidase are recommended when the spacer’s position is unsatisfactory but not pressing on critical organs.^11^ A scoping review on hyaluronidase use in managing aesthetic intervention complications showed high effectiveness in reversing HA filler nodules, with minimal adverse reactions.^12^

### Variations in technique

Some proceduralists from our institution were concerned that injecting the ‘full’ volume (eg, 9 mL) of Barrigel might raise the risk of infiltration—or at least raise the suspicion of infiltration on imaging. They suggested switching to smaller volumes (eg, 6 mL) to reduce the probability of these ambiguous findings and to avoid repeated queries about possible rectal wall infiltration. Although the reduced volume might decrease the risk of rectal wall infiltration, the reduced volume would also hypothetically reduce the intended dissection of the prostate from the rectum. Our proceduralists also noted difference between injection of HA (Barrigel) and the other commonly employed polyethylene glycol spacer gel (SpaceOAR), both of which are used at our institution. Hyaluronic acid can be injected with greater control and more slowly due the nature of the gel, which is inert and unchanged before and after injection. In contrast, the polyethylene glycol spacer gel is a compound of 2 liquid components that are loaded into a double barrel syringe and mixed upon injection. Due to the rapid conversion into gel form, it is recommended to inject the polyethylene glycol spacer gel within 10 to 15 seconds before it sets. Although this more rapid push may create a more uniform lift and separation of the prostate from the rectum, there is an inherent decreased element of manual control. Finally, it should be noted that all patients are slightly different in body habitus, natural spacing between the rectum and prostate, size of the prostate, and presence of adhesions or fibrosis between the rectum and prostate from prior surgeries, procedures, or infections. Guidelines and group input for product-specific selection, injection technique, and injected volume could therefore improve diagnostic accuracy through the minimization of radiographic variances.

### Spacer injection challenges

Injection of spacer gel between the prostate and rectum can be impeded by adhesions that can result from acute inflammation or chronic scarring between the prostate and rectum. This may restrict the ability for gel to flow evenly into the space. Possible reasons for chronic scarring include remote history of prostatitis, sequela of local regional inflammation from prior prostate surgery or radiation, or sequela of prior transrectal prostate biopsies. Possible reasons for acute adhesions or inflammation include active prostatitis and recent transrectal prostate biopsy or surgery. Whether the adhesions are acute or chronic, identification on cross-sectional imaging is not possible. Furthermore, the ability for the gel to evenly flow between the prostate and the rectum is only ascertained upon injection of the gel. The benefit of using HA in patients with adhesions between the prostate and rectum is that HA is an inert gel. As such, it can be slowly injected to ascertain the presence and degree of adhesions. Furthermore, the needle can gently be maneuvered and repositioned during injection through the space between the rectum and prostate to attempt to improve distribution. Lastly, the operator has discretion to decrease the quantity of HA injected if there are technical concerns for high risk of dissection into the rectum. Indeed, in our experience, we have decreased the amount of injection to mitigate risks when such adhesions are encountered and find this to be a sound, safe practice and, despite the decreased volume, can still provide some benefit for radiation safety.

### Need for consensus

Our institutional experience has highlighted the limited existing evidence on specific diagnostic and management strategies for rectal spacer infiltration, particularly in borderline cases or those with very small amounts of rectal infiltration. In this setting, given the low rate of severe complications, there is a need for prospective, multi-institutional data collection to guide best practices. Collection of such data could inform consensus guidelines and thus lead not only to decreased diagnostic error but also improve consistency of management approaches at large.

## Conclusion

Rectal **spacers** can provide meaningful reductions in rectal dose exposure and low-grade rectal complications, although their use is not without risk or controversy.^13^ Rectal wall infiltration remains a diagnostic and therapeutic challenge. Our findings emphasize the importance of multidisciplinary collaboration to improve diagnostic accuracy, optimize management strategies, and mitigate the risks associated with spacer placement. We emphasize the need for postinjection MRI to evaluate for rectal wall infiltration. Our experience demonstrates that not every rectal wall indentation is synonymous with infiltration and not every infiltration requires active management with injection of reversal agent. However, the ability to reverse HA spacers with hyaluronidase offers an important tool for clinicians in select cases and may prove to be the safest course of action before RT depending on the amount of infiltration, location of infiltration in reference to the intended RT, and type of RT. Further experience will be helpful to refine best practices for rectal spacer use to ensure that the benefits of spacer injections continue to outweigh the potential risks while maintaining optimal oncologic and quality-of-life outcomes for patients.

## Disclosures

Steven J. Frank reports grants from Eli Lilly and Elekta; grants and personal fees from C4 Imaging and Hitachi; personal fees from Varian, Boston Scientific, and the National Comprehensive Cancer Network (NCCN); and other support from Breakthrough Chronic Care, all outside the submitted work.
